# PKR Transduces MDA5-Dependent Signals for Type I IFN Induction

**DOI:** 10.1371/journal.ppat.1005489

**Published:** 2016-03-03

**Authors:** Alissa M. Pham, Felicia Gilfoy Santa Maria, Tanaya Lahiri, Eugene Friedman, Isabelle J. Marié, David E. Levy

**Affiliations:** Departments of Pathology and Microbiology and NYU Cancer Institute, NYU School of Medicine, New York, New York, United States of America; University of California, Santa Barbara, UNITED STATES

## Abstract

Sensing invading pathogens early in infection is critical for establishing host defense. Two cytosolic RIG-like RNA helicases, RIG-I and MDA5, are key to type I interferon (IFN) induction in response to viral infection. Mounting evidence suggests that another viral RNA sensor, protein kinase R (PKR), may also be critical for IFN induction during infection, although its exact contribution and mechanism of action are not completely understood. Using PKR-deficient cells, we found that PKR was required for type I IFN induction in response to infection by vaccinia virus lacking the PKR antagonist E3L (VVΔE3L), but not by Sendai virus or influenza A virus lacking the IFN-antagonist NS1 (FluΔNS1). IFN induction required the catalytic activity of PKR, but not the phosphorylation of its principal substrate, eIF2α, or the resulting inhibition of host translation. In the absence of PKR, IRF3 nuclear translocation was impaired in response to MDA5 activators, VVΔE3L and encephalomyocarditis virus, but not during infection with a RIG-I-activating virus. Interestingly, PKR interacted with both RIG-I and MDA5; however, PKR was only required for MDA5-mediated, but not RIG-I-mediated, IFN production. Using an artificially activated form of PKR, we showed that PKR activity alone was sufficient for IFN induction. This effect required MAVS and correlated with IRF3 activation, but no longer required MDA5. Nonetheless, PKR activation during viral infection was enhanced by MDA5, as virus-stimulated catalytic activity was impaired in MDA5-null cells. Taken together, our data describe a critical and non-redundant role for PKR following MDA5, but not RIG-I, activation to mediate MAVS-dependent induction of type I IFN through a kinase-dependent mechanism.

## Author Summary

Induction of type I interferon (IFN) during viral infection is a key step in the activation of innate host defense. Critical to this response is the ability to recognize viral nucleic acids in the host cell cytoplasm. Detection of viral RNA is mediated by RNA helicases, including RIG-I and MDA5, which sense 5’-phosphorylated RNA and long double-stranded viral RNA, respectively. The importance of the IFN response is underscored by the variety of mechanisms through which viruses circumvent signaling by inactivating components of this pathway, including targeting the cellular kinase, protein kinase R (PKR), to evade host defenses. Activation of PKR by virus infection is known to result in an overall block of host translational machinery. Here, we showed that a function of PKR, independent of translation inhibition, was critical for IFN production downstream of MDA5, but not RIG-I. In addition, MDA5 was required for the stimulation of PKR catalytic activity that occurred in response to infection by an MDA5-restricted virus, but not in response to a RIG-I-dependent virus. These findings identified a previously uncharacterized role for PKR catalytic function that cooperates with MDA5 signaling and highlights an unexpected role for MDA5 in stimulating the enzyme activity of PKR.

## Introduction

The innate immune response allows for the rapid production of type I interferons (IFNs) and other proinflammatory cytokines to counteract invading viral pathogens. This response relies, in part, on a group of molecules collectively referred to as pattern recognition receptors (PRRs), which recognize pathogen-associated molecular patterns generated during the course of infection. The detection of virus infection is mediated primarily by cytoplasmic sensors for both RNA and DNA, which include members of the RIG-like helicase (RLH) family for RNA detection and a variety of cytoplasmic proteins for detection of DNA [1].

To date, there are three members of the RLH class of PRRs, all of which are cytosolic RNA helicases that recognize double-stranded RNA (dsRNA): retinoic acid-inducible gene I (RIG-I) [2], melanoma differentiation-associated gene 5 (MDA5) [3] and laboratory of genetics and physiology-2 (LGP2) [4]. The RLH proteins belong to a family of DExD/H box-containing RNA helicases, and in addition, RIG-I and MDA5 possess two N-terminal caspase recruitment and activation domains (CARDs), and a C-terminal regulatory domain [2, 3]. Despite this homology, each sensor displays a different affinity for distinct dsRNA ligands and, hence, different viruses [5]. RIG-I, the most extensively studied member of the RLH family, recognizes short dsRNA segments bearing 5' triphosphate structures [6–9], whereas MDA5 recognizes long dsRNA that likely harbor higher-ordered RNA structures [10, 11]. LGP2, unlike RIG-I and MDA5, lacks the N-terminal CARD domains necessary for activating downstream signaling components and was initially identified as a negative regulator of RIG-I [4, 12]. However, more current evidence suggests that LGP2 may instead be a positive regulator of both RIG-I and MDA5 [13, 14].

Although RIG-I and MDA5 recognize different dsRNA motifs, both PRRs converge on a single adaptor protein to stimulate a signaling cascade inducing IFN [15, 16]. This adaptor protein (designated MAVS throughout this work) is known variously as IFNβ promoter stimulator 1 (IPS-1) [17], mitochondrial antiviral signaling protein (MAVS) [18], virus-induced signaling adaptor (VISA) [19] or CARD adaptor inducing IFNβ (CARDIF) [20]. MAVS contains a single CARD, a central proline-rich region and a C-terminal hydrophobic region that anchors the protein to the outer mitochondrial membrane [21]. Upon activation, RIG-I or MDA5 bind MAVS via CARD-CARD interactions and ubiquitin chains, resulting in MAVS aggregation on the mitochondrial membrane [22]. Aggregated MAVS provides a platform for recruitment of signaling molecules leading to activation of transcription factors critical for IFN gene induction, including NFκB, ATF2/c-jun and members of the interferon regulatory factor (IRF) family [23]. Among the MAVS-recruited adaptors are members of the tumor necrosis factor (TNF) receptor-associated factor (TRAF) family members, specifically TRAF2, TRAF3 and TRAF6 [19, 24, 25]; which transmit signals downstream to the inhibitor of NFκB kinase (IKK) complex to drive nuclear translocation of NFκB [20, 26]. Additionally, MAVS signals to TRAF family member-associated NFκB activator (TANK) binding kinase 1 (TBK1) and IKKε, to phosphorylate IRF3 and IRF7, and induce transcriptional activation of IFN and other proinflammatory cytokines.

IFN can signal in an autocrine or paracrine manner to trigger induction of hundreds of IFN-stimulated genes (ISGs) that act to fortify host defenses. Of note is PKR, an ISG whose activity is triggered by the presence of foreign dsRNA. PKR was initially identified for its ability to inhibit viral protein translation in vaccinia virus (VV)-infected cells [27]. PKR is a serine/threonine kinase that contains two domains, a N-terminal regulatory dsRNA binding domain, and a C-terminal catalytic domain [28, 29]. Although PKR expression is inducible by IFN, it is also expressed at a basal level in most cells as an inactive monomer. Latent PKR must be activated to be functional, which is achieved by binding dsRNA or by a variety of other stimuli. Once activated, PKR undergoes a dimerization-dependent conformational change and autophosphorylation, stimulating its catalytic activity toward protein substrates, the best characterized being eukaryotic initiation factor 2 α-subunit (eIF2α) [29]. Phosphorylation of eIF2α results in inhibition of protein translation.

Although inhibition of translation is the best characterized function for PKR, it has been implicated in additional cellular responses, including apoptosis and autophagy [30]. Mounting evidence also suggests a role for PKR in the production of type I IFN and other proinflammatory cytokines [31]. PKR may play a role in the activation of NFκB, a transcription factor required for IFN induction, presumably via its interaction with the IKK complex [32]. Additionally, PKR has also been shown to interact with several members of the TRAF family, including TRAF2 and TRAF6 [33], two proteins involved in MAVS signaling. Moreover, a number of studies have shown that PKR is involved in IFN induction in response to polyriboinosinic:polyribocytidylic acid (pIC), a dsRNA mimetic [34–36]. PKR may also be important for IFN induction in response to some, but not all, viral infections [37–40].

Here, we report evidence that PKR is required for type I IFN induction in response to viruses recognized by MDA5. This role for PKR was independent of its canonical function in translation inhibition and phosphorylation of the initiation factor eIF2α, but nonetheless, dependent on its catalytic activity. Induction of IFN in this MDA5-PKR-dependent manner occurred through IRF3 activation, and likely involved a physical interaction between PKR and MDA5. Furthermore, PKR activation by virus was impaired in cells lacking MDA5, and studies involving artificial PKR activation in the absence of virus infection demonstrated IRF3 phosphorylation and IFN expression in a MAVS-dependent, but MDA5-independent fashion. Taken together, these results suggest MDA5 and PKR cooperate to transduce dsRNA-dependent induction of type I IFN production.

## Results

### Vaccinia virus lacking E3L stimulates IFN production through a pathway requiring MDA5, but not RIG-I

**Fig 1:**
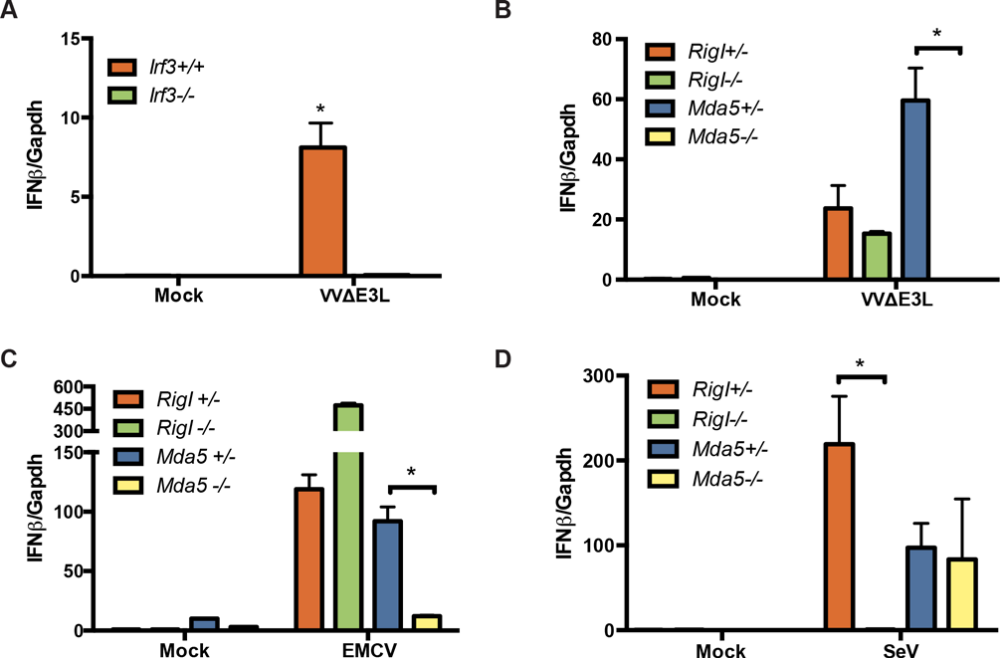
VVΔE3L signals through IRF3 and MDA5, but not RIG-I. (A) Wild type (WT) and *Irf3* ^*-/-*^ mouse embryonic fibroblasts (MEFs) were infected with vaccinia virus lacking E3L (VVΔE3L) (MOI = 3) for 8 h and IFNβ expression was measured by quantitative real time PCR (qRT-PCR). (B) Infection of *RigI* ^*-/-*^ and *Mda5* ^*-/-*^ MEFs and corresponding WT counterparts with VVΔE3L (MOI = 3) for 8 h. IFNβ expression was quantified by qRT-PCR. (C) Same as in (B) for encephalomyocarditis virus infection (EMCV) (MOI = 3). (D) Same as in (B) following Sendai virus (SeV) (100 HA units/mL) infection. Data shown represent means and standard deviations of three independent experiments. Statistically significant differences (p< 0.001) are indicated (*).

Vaccinia virus (VV), like all members of the *Orthopoxvirus* family, encodes multiple virulence determinants that function by impairing host innate immune responses. In particular, the E3L protein is capable of sequestering viral RNA and inhibiting PKR activation, thus blocking a critical component of IFN-dependent antiviral responses [41]. To assess IFN induction in the absence of this inhibitor, we measured IFNβ expression in cells following infection with a mutant virus lacking this gene, VVΔE3L. Infection of WT (WT) mouse embryo fibroblasts (MEF) with VVΔE3L resulted in abundant expression of IFNβ (Fig 1A). As expected, IFNβ expression was completely dependent on the presence of the transcription factor IRF3, as shown by the lack of IFNβ expression following infection of *Irf3* ^-/-^ cells. In an effort to identify the sensor mediating the innate response to VVΔE3L, the expression of IFNβ was measured in the absence of the cytosolic RNA sensors, MDA5 and RIG-I (Fig 1B). In parallel with a known stimulator of MDA5, encephalomyocarditis virus (EMCV) (Fig 1C), VVΔE3L induction of IFN was also dependent on MDA5 (Fig 1B). Interestingly, the alternative RNA sensor, RIG-I, was superfluous for IFNβ expression in response to VVΔE3L infection, since IFNβ expression was normal in infected *RigI* ^-/-^ cells. As expected, IFNβ expression in cells infected by Sendai virus (SeV), a negative-sense RNA virus, showed distinct sensor dependence. SeV-infected cells expressed IFNβ in the absence of MDA5, but this response was undetectable in the absence of RIG-I (Fig 1D). Therefore, VVΔE3L appears to produce ligands that are recognized by MDA5, but not by RIG-I, in the absence of the E3L protein, consistent with previous reports suggesting MDA5-dependent inflammatory responses to VV [42].

### PKR is required for IFN induction by vaccinia virus

**Fig 2:**
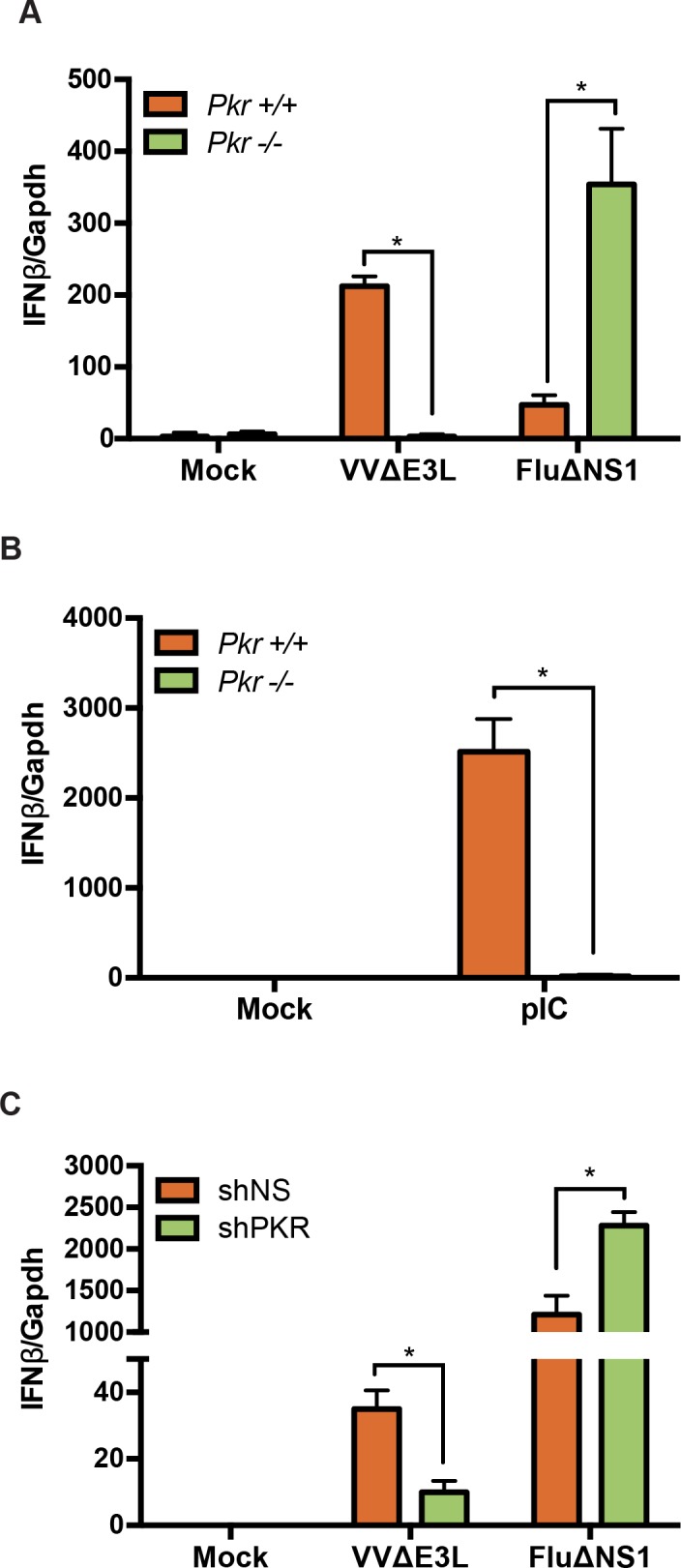
PKR is required for VVΔE3L-mediated IFNβ induction. (A) Infection of WT or *Pkr* ^*-/-*^ MEFs with VVΔE3L (MOI = 3) or FluΔNS1 (MOI = 1) for 8 h followed by quantifying IFNβ expression by qRT-PCR. (B) Treatment of WT and *Pkr* ^*-/-*^ MEFs with polyriboinosinic:polyribocytidylic acid (pIC) for 4 h followed by analysis of IFNβ expression by qRT-PCR. (C) A549 shNS (nonspecific) and A549 shPKR cells were infected with VVΔE3L or FluΔNS1 for 8 h and assayed for IFNβ mRNA by qRT-PCR. Data represents the mean and standard deviation from triplicate samples. Significance (*) was determined by an unpaired t-test (p< 0.001).

Vaccinia virus E3L protein and influenza virus NS1 protein are both virulence factors that, due to their affinity for dsRNA, have the ability to inhibit PKR activity [43–47]; infection in their absence efficiently triggers PKR function. Since E3L can abrogate IFN induction [35] and PKR is one of its major targets, we investigated the role of PKR in the cellular response to infection with the VV mutant, VVΔE3L. We infected WT and PKR-null MEF cell lines with VVΔE3L or influenza A virus lacking the NS1 protein (FluΔNS1). Infection of WT MEFs with either VVΔE3L or FluΔNS1 resulted in the induction of IFNβ mRNA. (Fig 2A) However, PKR-null MEFs demonstrated a significant impairment in IFNβ mRNA levels in response to VVΔE3L, but not FluΔNS1 infection. In fact, the absence of PKR boosted IFNβ levels in response to FluΔNS1 infection, possibly due to positive feedback that is otherwise prevented when protein synthesis is inhibited by activated PKR [48].

PKR activity is triggered during viral infection by interaction with dsRNA, which can be mimicked by transfection of pIC [29]. Consistent with previous reports [49, 50], cells lacking PKR showed a significant reduction in pIC-induced IFN compared to WT MEFs (Fig 2B). We also assayed the role of PKR in human cell lines by stably reducing its expression in A549 lung adenocarcinoma cells with a short hairpin RNA (A549 shPKR; S1 Fig). Consistent with results in PKR-null MEFs, A549 shPKR cells showed significantly lower levels of IFNβ mRNA following infection with VVΔE3L compared to A549 cells expressing a non-specific shRNA (A549 shNS; Fig 2C). However, levels of IFNβ following FluΔNS1 infection were not impaired in the A549 shPKR cells. Similar to the result with FluΔNS1 in PKR-null MEFs (Fig 1A), IFNβ was induced and even enhanced in response to FluΔNS1 infection following PKR knockdown (Fig 2C).

### PKR catalytic activity, but not eIF2α phosphorylation, is required for IFN induction

**Fig 3:**
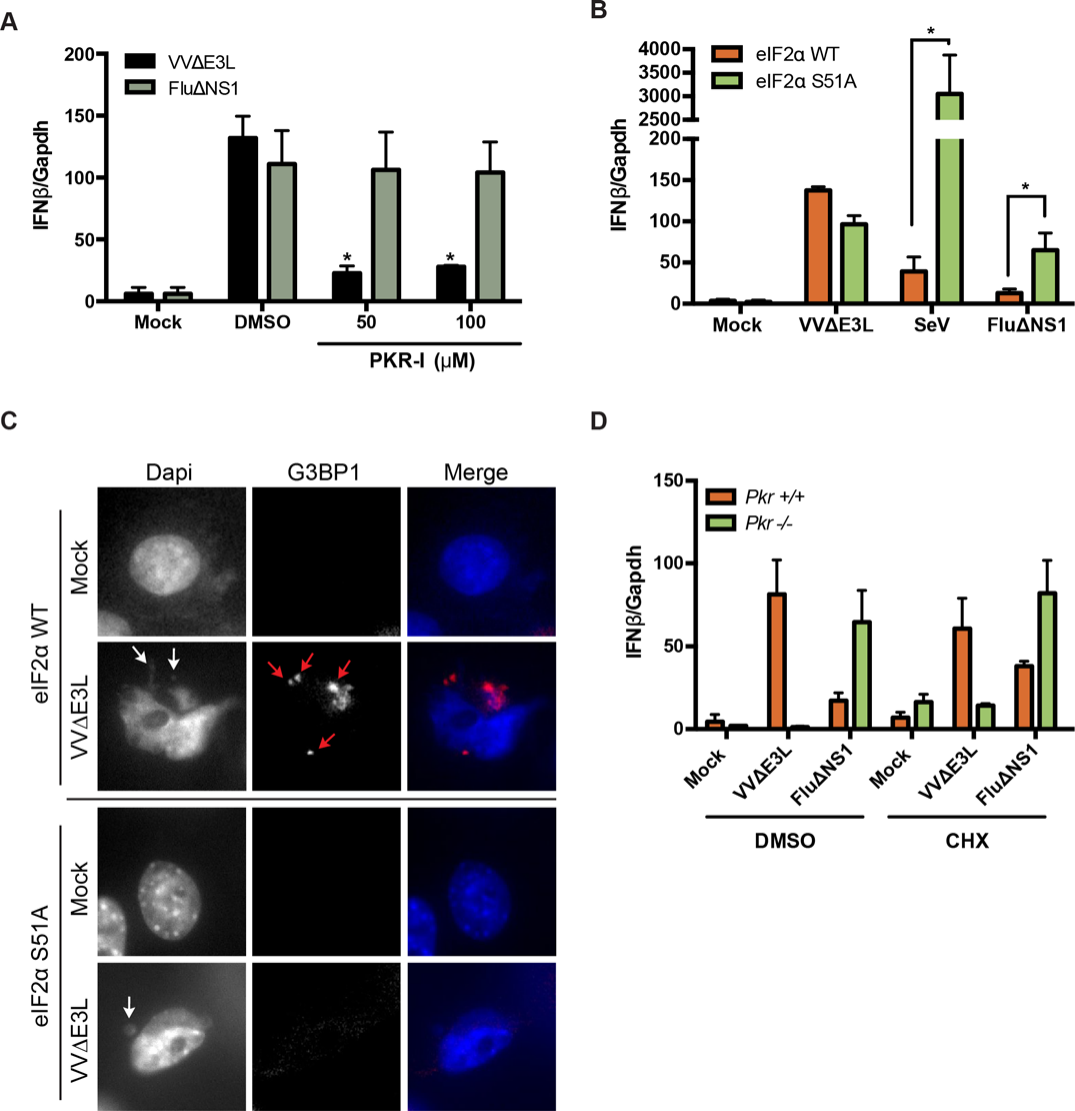
PKR kinase activity, but not eIF2α phosphorylation, translation inhibition, or stress granule formation, is required for IFNβ induction. (A) WT MEFs were treated with the indicated concentrations of PKR inhibitor (PKR-I) or DMSO and infected with VVΔE3L (MOI = 3) or FluΔNS1 (MOI = 1) for 8 h prior to quantifying IFNβ expression. (B) WT or eIF2α-S51A MEFs were infected with VVΔE3L (MOI = 3), FluΔNS1 (MOI = 1), or SeV (100 HA units/mL) for 8 h prior to quantifying IFNβ expression. (C) WT or eIF2α-S51A MEFs uninfected (mock) or infected with VVΔE3L were stained with DAPI and antibodies against G3BP1 to detect viral DNA and stress granules, respectively. White arrows indicate cytoplasmic DNA (likely representing poxvirus replication centers; red arrows indicate G3BP1-positive stress granules. (D) WT or PKR-null MEFs were infected with VVΔE3L (MOI = 3) or FluΔNS1 (MOI = 1) for 8 h. Cells were treated with DMSO or 10 ug/ml cycloheximide (CHX) for the final 4 h of infection followed by qRT-PCR analysis for IFNβ gene expression.

Impaired IFN induction in PKR-null and PKR-knockdown cells during VVΔE3L infection indicated a key role for this enzyme in mediating this response. To assess whether the catalytic function of PKR was required, we treated cells with a PKR inhibitor (PKR-I) that prevents RNA-induced PKR autophosphorylation [51], and measured IFNβ induction following VVΔE3L infection. WT MEFs were treated with PKR-I and infected with either VVΔE3L or FluΔNS1. Both viruses induced substantial expression of IFNβ in vehicle-treated cells (DMSO) that was approximately 100-fold greater than uninfected samples (Fig 3A). PKR-I treatment impaired IFNβ mRNA levels in a dose-dependent manner following infection with VVΔE3L, but not FluΔNS1. This result demonstrated that PKR catalytic activity contributed to IFNβ induction.

A critical component of the antiviral action of PKR is derived from inhibition of host protein translation due to the phosphorylation of the eukaryotic translation initiation factor 2 α-subunit (eIF2α) [52]. To assess the contribution of this activity to IFNβ induction during VVΔE3L infection, we examined responses in a cell line that contains a mutation in eIF2α at serine 51 (eIF2α S51A). This mutation blocks its ability to be phosphorylated by eIF2α kinases [53] and renders cells resistant to the translational inhibitory effects of PKR [54]. Interestingly, this mutant cell line showed no deficit in VVΔE3L-induced IFNβ mRNA levels (Fig 3B), indicating that eIF2α phosphorylation and, presumably, PKR-mediated protein translation shutoff were not involved in PKR-mediated IFN induction. Similarly, there was no deficit in IFN induction following infection with the RIG-I-activating viruses SeV and FluΔNS1. In fact, both viruses induced greater levels of IFN in the absence of eIF2α phosphorylation (Fig 3B), mimicking the heightened induction of IFN observed in response to these viruses in PKR-null and PKR-knockdown cells (Fig 2A and 2C).

Phosphorylation of eIF2α leads to formation of stress granules in virus-infected cells, a process that appears to contribute to innate antiviral immunity [55]. Stress granules contain stalled protein translational machinery, due at least in part to translational inhibition from eIF2α inactivation. To determine if stress granule formation due to eIF2α phosphorylation by PKR explained the requirement for PKR during VVΔE3L infection, we probed WT and eIF2α S51A cells for stress granules, using an antibody against the stress granule marker, G3BP1 [56]. As shown in Fig 3C, WT cells displayed stress granule formation in response to infection (upper panel). They also displayed cytoplasmic DAPI-staining bodies that likely represent vaccinia poxvirus factories [57]. In contrast, S51A cells failed to display G3BP1-positivity, although DAPI-staining poxvirus factories were present in infected cells (Fig 3C, lower panel). Approximately 64% of infected WT cells displayed stress granules, while examination of multiple fields of infected mutant cells failed to detect any G3BP1-positive cytoplasmic granules. This observation is consistent with previous reports that virus-induced stress granule formation is dependent on eIF2α S51 phosphorylation [58]. Coupled with the observation that eIF2α S51A cells produced IFN in response to infection (Fig 3B), these data suggest that stress granule formation during VVΔE3L infection is not required for IFN production.[55]

To further confirm that host translational shutoff was not the attribute of PKR activation that was required for IFN induction, we infected WT and PKR-null MEFs with VVΔE3L or FluΔNS1 while inhibiting translation pharmacologically with cycloheximide (CHX). If the inability of PKR-null MEFs to shut off translation was causing the deficit in IFN induction, as has been demonstrated for other viruses [59], then CHX treatment to induce PKR-independent translational arrest might be expected to rescue IFN induction in the absence of PKR. We tested this notion by comparing IFNβ induction in VVΔE3L-infected PKR-deficient cells in the presence and absence of CHX. However, despite elevated basal levels of IFNβ mRNA following CHX treatment that probably reflects mRNA stabilization [60], VVΔE3L infection was still incapable of inducing IFNβ in PKR-null MEFs (Fig 3D). Taken together, these data suggest that although PKR and its catalytic function are required for VVΔE3L-induced IFN production, its canonical antiviral effector functions involving eIF2α phosphorylation, translational inhibition, and subsequent stress granule formation are not involved.

### PKR is required for VVΔE3L-induced IRF3 nuclear translocation

**Fig 4:**
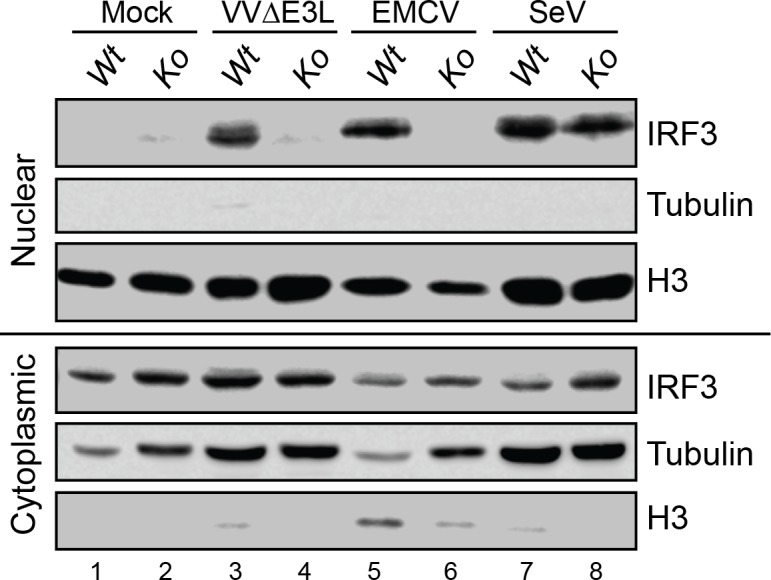
PKR is required for IRF3 activation during VVΔE3L infection. WT and *Pkr* ^*-/-*^ MEFs were infected with the indicated viruses (VVΔE3L and EMCV: MOI = 3, SeV: 200 HA units/mL) for 8 h. IRF3 nuclear translocation and total IRF3 expression were measured by western blotting of nuclear and cytoplasmic extracts. Detection of histone H3 and tubulin were used as loading controls for nuclear and cytoplasmic compartments, respectively. The data shown are representative of three independent experiments.

Our data suggest that PKR plays an important role in IFN induction in response to VVΔE3L infection or pIC treatment (Fig 2B), but not FluΔNS1 (Fig 2A). Since IRF3 is required for the induction of IFNβ [61, 62], we asked whether its activation was impaired during infection of cells lacking PKR. To this end, WT and PKR-null MEFs were infected with a panel of viruses (Fig 4), and examined for IRF3 nuclear translocation as an indication of activation. Consistent with data for FluΔNS1 (Fig 2A and 2C), which displayed no dependency on PKR for IFN production, infection of PKR-null MEFs with another RIG-I agonist and strong inducer of IRF3 phosphorylation, Sendai virus (SeV), did not result in a significant defect in IRF3 activation (Fig 4). In contrast, IRF3 activation in response to either VVΔE3L or EMCV, both MDA5 stimulators, was abrogated in the absence of PKR, as indicated by the inability to detect nuclear IRF3 in extracts from cells infected with these viruses (Fig 4). PKR-null cells were infected at least as well as WT cells, as assayed by quantitation of viral RNA following infection (S2 Fig), confirming that failure to induce IRF3 translocation was not due to a defect in infectivity of PKR-null cells. Taken together, these data place failure to activate IRF3, a necessary component of IFN induction (Fig 1A), as a proximal cause of impaired IFNβ induction in the absence of PKR.

### PKR is involved in MDA5, but not RIG-I signaling

**Fig 5:**
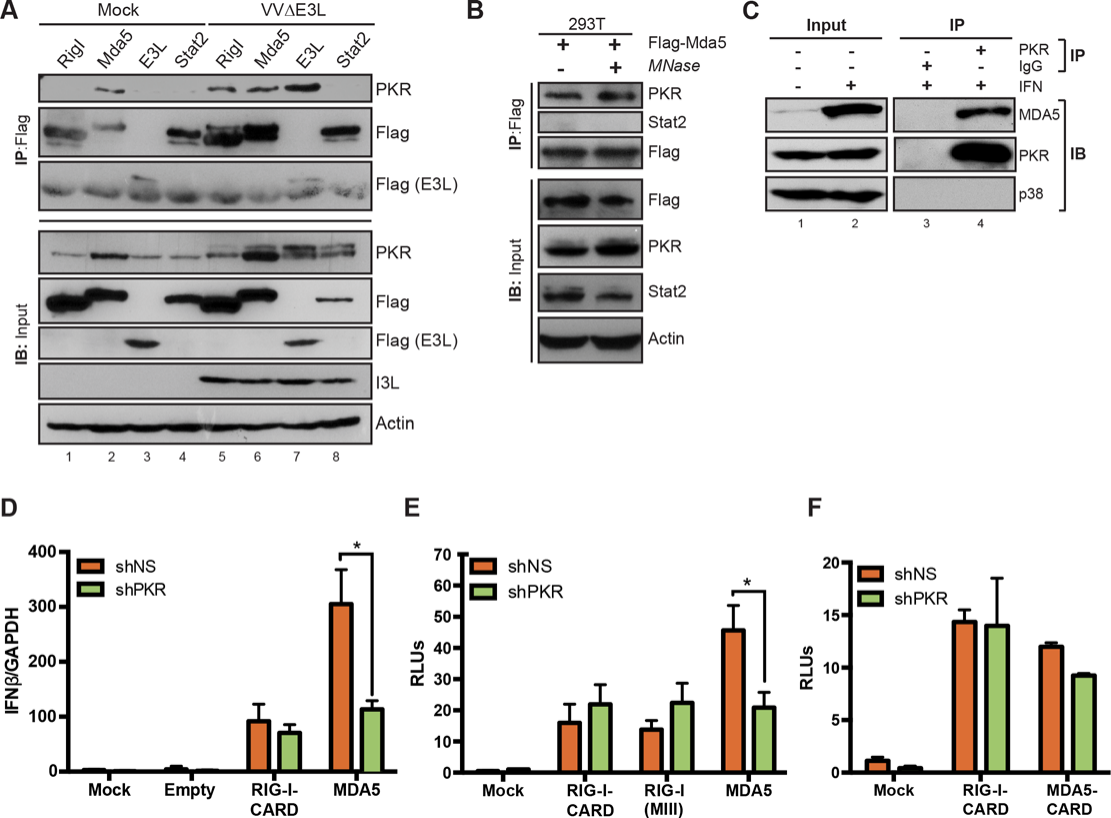
Interaction between MDA5 and PKR. (A) HEK293T cells were transfected with the indicated flag-tagged plasmids, infected with VVΔE3L (MOI = 3) for 8 h, and analyzed by immunoprecipitation and immunoblotting, as indicated. (B) Extracts from HEK293T cells transfected with flag-tagged MDA5 were untreated or treated with micrococcal nuclease (MNase) prior to flag immunoprecipitation and immunoblotting. (C) Extracts from A549 cells untreated or treated with IFN-α (100 u/ml for 8 h) were analyzed by immunoprecipitation and immunoblotting, as indicated. Input levels represent 10% of the cell lysates used for immunoprecipitation. (D) A549 cells expressing shNS or shPKR constructs were transduced with lentiviruses expressing constitutively active truncated RIG-I (RIG-I-CARD) or full-length MDA5, and analyzed for IFNβ expression 48 h post transduction by qRT-PCR. (E) IFNβ luciferase activity of HEK293T cells transiently expressing RIG-I-CARD, constitutively active full length RIG-I (RIGI-MIII), and MDA5, along with IFNβ-luc and βGal reporter plasmids. Cells were analyzed 48 h post transfection. (F) IFNβ luciferase activity was measured as in (E) for HEK293T cells transfected with constructs expressing the CARD domains of either RIG-I (RIG-I-CARD) or MDA5 (MDA5-CARD). shNS = short-hairpin against non-specific RNA; shPKR = short-hairpin against PKR mRNA; RLU = relative luciferase units. Significant differences (p< 0.01) are indicated (*).

The requirement for PKR in IFN induction by the various viruses and treatments we tested correlated with the known involvement of MDA5 in signaling. Specifically, the ability of the tested agonists to induce IRF3 phosphorylation or IFNβ expression in the absence of PKR fell into two distinct categories based on the PRR responsible for their recognition: those that require RIG-I for IFN induction, FluΔNS1 and SeV [5], were PKR independent, while those that require MDA5, VVΔE3L (Fig 1B), EMCV and pIC [63] were PKR-dependent. Involvement of PKR downstream of MDA5 prompted us to investigate the possibility of direct protein interactions. HEK293T cells were transfected with FLAG-tagged versions of RIG-I, MDA5, the E3L protein from VV, a known PKR-interacting protein, or STAT2, a cytoplasmic signaling and transcription factor with no known connection to PKR [64]. Transfected cells were infected with VVΔE3L, and protein interactions were detected by co-immunoprecipitation. Immunoprecipitation with anti-FLAG antibodies and probing for PKR showed co-purification of endogenous PKR with RIG-I, MDA5, as well as E3L, but not with STAT2 (Fig 5A). The PKR interaction with MDA5 occurred in both uninfected as well as VVΔE3L-infected cells. Interestingly, the interaction between PKR and either RIG-1 or E3L depended on VVΔE3L infection, while PKR interaction with MDA5 did not follow this trend (Fig 5A).

Because both MDA5 and PKR are RNA-binding proteins, we explored whether this protein-protein interaction was dependent on nucleic acid. To this end, extracts from transfected cells were incubated with micrococcal nuclease prior to immunoprecipitation. MDA5 and PKR co-immunoprecipitated regardless of nuclease treatment (Fig 5B), indicating that their interaction was not bridged by RNA.

To extend these results, we tested the interaction between endogenous proteins in human A549 cells (Fig 5C). Since MDA5 is a low abundance protein, we induced its levels by pretreatment of cells with IFN (Fig 5C, lanes 1 and 2). Under these conditions, immunoprecipitation of endogenous PKR effectively purified endogenous MDA5 (lane 4), while control purifications using non-specific IgG recovered neither protein (lane 3). The abundant cellular protein p38 MAPK served as a control for non-specific binding and was not recovered by either antibody treatment. Together with the overexpression studies, these results confirm that MDA5 and PKR associate in a complex independent of viral infection and RNA binding.

To directly assess a requirement for PKR downstream of RIG-I and MDA5, we tested signaling from ectopically expressed and constitutively active RLH proteins. Full-length MDA5, which is sufficient to induce IFN when over-expressed, or a constitutively-active RIG-I mutant expressing only the N-terminal CARD domains (RIG-I-CARD) [2], were transduced into A549 cells following PKR knockdown (S1 Fig), and levels of IFN mRNA were measured (Fig 5D). The expression of RIG-I-CARD induced comparable levels of IFNβ in the control A549 shNS and knockdown A549 shPKR cells (Fig 5D). However, MDA5 overexpression induced significantly lower levels of IFNβ in the A549 shPKR compared to the A549 shNS control cells. Together, these results are consistent with our previous observations that PKR was required for IFNβ induction by MDA5, but not by RIG-I ligands. Comparable levels of RIG-I-CARD and MDA5 were transduced into both cell lines, as judged by expression of the linked IRES-mCherry marker (S3 Fig).

To further determine whether PKR dependence of MDA5-induced IFN expression was also observed at the level of transcriptional activation, activation of an IFNB-luciferase reporter was measured in response to the constitutively active constructs (Fig 5E). Transfected HEK293T cells depleted for PKR (S4 Fig) displayed reduced activation of an IFNβ reporter in response to expression of MDA5. A potential caveat to the conclusion that PKR mediates MDA5, but not RIG-I signaling, could be that the constitutively active RIG-I construct lacked the RNA-binding, helicase, and regulatory regions of the protein, while the analogous domains were intact in the MDA5 construct. To address whether non-CARD domain regions could mediate an RLR-PKR connection, we interrogated the role of PKR downstream of full-length RIG-I. To this end, we ectopically expressed a constitutively active full-length RIG-I mutant that retains RNA binding ability [65]. In contrast, but consistent with results in Fig 5D, both RIG-I-CARD and mutated full-length RIG-I mediated induction of IFNβ regardless of the presence or absence of PKR. Similarly, we tested whether the dependence of MDA5 on PKR involved its RNA-helicase domain. Cells were transfected with truncated MDA5 expressing only the CARD domains (MDA5-CARD), analogous to the RIG-I-CARD construct. Interestingly, MDA5-CARD induced IFN expression in a similar manner to RIG-I-CARD, regardless of the presence or absence of PKR (Fig 5F). These data suggest a critical dependence of MDA5 signaling on PKR that is mediated by its RNA helicase domain and is distinct from the action of RIG-I.

### PKR activation is sufficient to induce IFN

**Fig 6:**
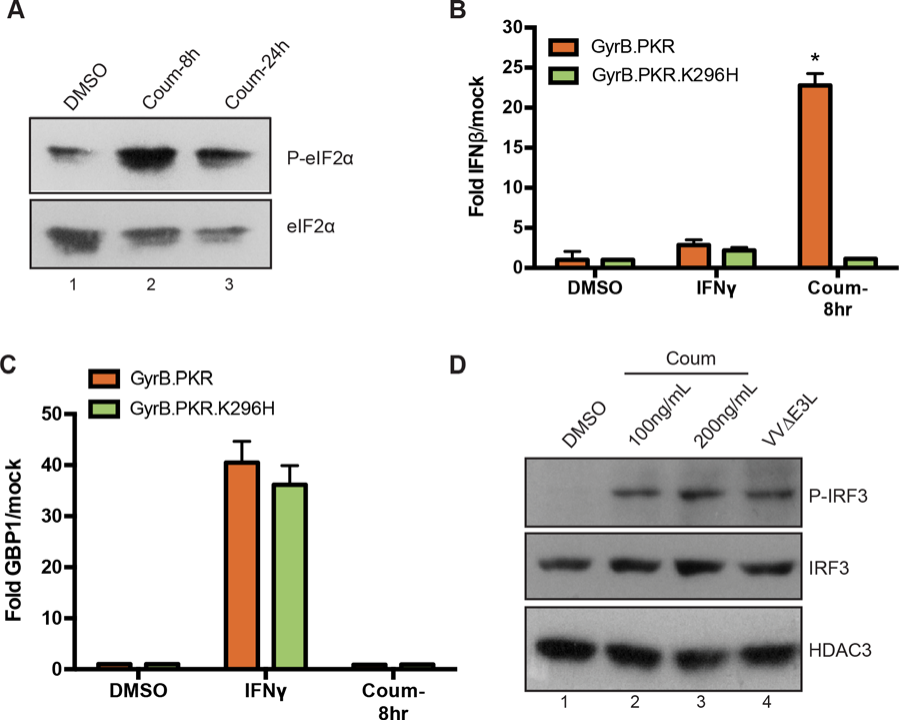
Ectopic PKR activation induces IFNβ expression in the absence of viral infection. (A) HT1080-GyrB-PKR cells were treated with 100 ng/ml coumermycin (coum) for 8 or 24 h, followed by western blot analysis for phospho-eIF2α. (B, C) HT1080-GyrB-PKR and HT1080-GyrB-PKR-K296H cells were treated with coumermycin or IFNγ for 8 h followed by qRT-PCR analysis to quantify expression of IFNβ (B) or GBP1 (C). Significant difference (p< 0.001) is indicated (*). (D) HT1080-GyrB-PKR cells were treated either with coumermycin (8 h) or infected with VVΔE3L (6 h), then assayed for IRF3 phosphorylation by western blotting. Detection of total IRF3 and HDAC3 indicated equal protein recovery.

To directly assess the ability of PKR to induce IFN, we activated PKR catalytic function in the absence of viral infection. Fusing the PKR catalytic domain (residues 258–551) to the dimerization domain of *E*. *coli* Gyrase B (GyrB) protein (GyrB.PKR) creates a conditionally active version of the kinase that responds to coumermycin treatment [66, 67]. In this context, GyrB, which dimerizes in the presence of coumermycin, replaces the dsRNA-binding domain that normally regulates PKR. Treatment of HT1080-GyrB.PKR cells with coumermycin resulted in an increase in the levels of phosphorylated eIF2α after 8 hours (h) in the absence of viral infection (Fig 6A). Coumermycin treatment of HT1080-GyrB.PKR cells induced robust levels of IFNβ expression (Fig 6B), but IFNβ levels remained unaffected when coumermycin was used to dimerize a catalytically inactive form of GyrB.PKR (K296H) (Fig 6B, green bars), consistent with the requirement for intact PKR catalytic function (Fig 3A). Treatment with coumermycin induced IRF3 phosphorylation in a manner equivalent to VVΔE3L infection (Fig 6D), suggesting that activation of PKR is sufficient to activate IRF3. Additionally, coumermycin treatment induced the expression of the NFκB target genes, TNFα and IL1β (S5 Fig), consistent with previous reports linking PKR to NFκB activation [32]. However, coumermycin treatment had no effect on the expression of an IFNγ-induced gene, GBP1 (Fig 6C), which, unlike IFNβ, is dependent on STAT1 and IRF1 rather than IRF3 and NFκB [68].

### MAVS, but not MDA5, is essential for PKR-induced IFN production

**Fig 7:**
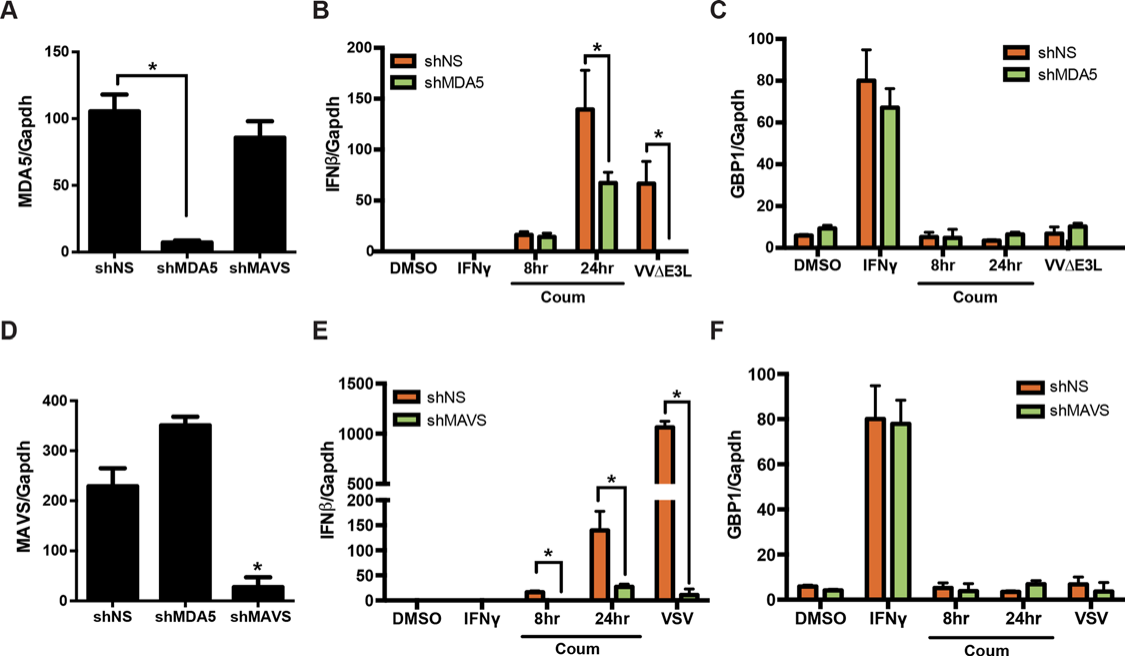
PKR-induced IFNβ expression requires MAVS. HT1080-GyrB-PKR cells expressing scrambled or shRNAs specific for MDA5 (A, B, C) or MAVS (D, E, F) were treated with coumermycin, IFNγ, or infected with VVΔE3L or VSV (MOI = 3) for 8 h, and scored by qRT-PCR either for MDA5, MAVS, IFNβ or GBP1 mRNA expression.

**Fig 8:**
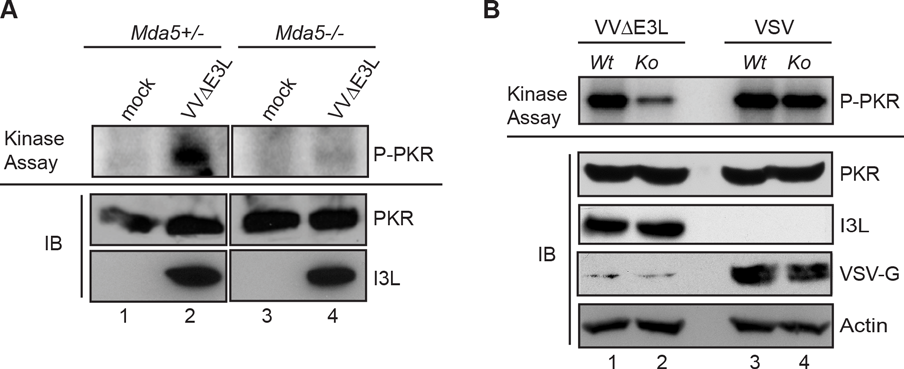
MDA5 enhances VVΔE3L-induced PKR catalytic activity. (A) *Mda5* ^*+/-*^ and *Mda5* ^*-/-*^ MEFs were infected with VVΔE3L and cell lysates were harvested at 8 h post infection. PKR immunoprecipitates were assessed for catalytic activity by *in vitro* kinase assay, and levels of phosphorylated PKR were quantified by phosphorimager (upper panel). Total PKR and VV I3L protein levels (lower panel) were detected by western blotting. (B) Extracts from cells infected with VVΔE3L or SeV were analyzed by *in vitro* kinase assay as in (A). Antibodies to I3L and VSV-G were used to document equal levels of virus infection in WT and KO cells.

These data indicated that PKR was necessary and sufficient for IFN production. To probe the epistatic relationship of PKR relative to other signaling components in this pathway, we silenced either MDA5 or MAVS in HT1080-GyrB.PKR cells and activated PKR catalytic function with coumermycin after 8 and 24 h (Fig 7). Silencing MDA5 (Fig 7A) had little effect on IFNβ induction in response to PKR activation after 8 h, but a reproducible difference was detected after 24 h of coumermycin stimulation (Fig 7B). Interestingly, MDA5 loss abrogated IFN induction in response to VVΔE3L infection, indicating that PKR function is epistatic to MDA5. In contrast, silencing MAVS (Fig 7D) largely abrogated the ability of PKR to induce IFN (Fig 7E), as well as impairing responses to viral infection, such as VSV. As expected, IFNγ-induced expression of GBP1 was unaffected by the knockdown of either MDA5 or MAVS (Fig 7C and 7F). That artificial activation of PKR was capable of inducing IFNβ in the absence of MDA5, but remained dependent on MAVS led us to conclude that it most likely acts during infection to transmit signals between these two elements of the pathway. While PKR may be capable of functioning independently from MDA5 when activated in an RNA-independent manner (Fig 7B, 8 h), its ability to signal to MAVS during viral infection is augmented in an MDA5-dependent manner, suggesting that PKR acts ‘downstream’ of MDA5 and ‘upstream’ of MAVS.

### PKR phosphorylation is impaired in the absence of MDA5

Since the catalytic activity of PKR is required for its ability to augment IFNβ induction in VVΔE3L infected cells (Fig 3), we assayed this function in response to infection to determine its dependence on MDA5. PKR was immunoprecipitated from VVΔE3L-infected MEFs in the presence or absence of MDA5, and the immunoprecipitates were assayed for catalytic activity following incubation with ^32^P-ATP *in vitro* (Fig 8A). Very little activity was detected in samples from uninfected cells (lanes 1 and 3), while kinase activity was significantly enhanced following infection of WT cells (lane 2). Importantly, little increase in activity was observed for PKR isolated from *Mda5* ^*-/-*^ cells following infection (lane 4, upper panel), in spite of equal quantities of total protein being recovered from all cells (lower panel). Quantification of multiple independent experiments demonstrated consistently and significantly reduced induction of PKR catalytic activity in *Mda5* ^*-/-*^ relative to WT cells following infection. To determine if impairment of PKR activation was an intrinsic property of MDA5-null cells or was restricted to viruses capable of activating MDA5, we infected WT and *Mda5* ^*-/-*^ cells with the RIG-I activating virus, vesicular stomatitis virus (VSV). VSV infection led to robust activation of PKR autophosphorylation regardless of MDA5 expression (Fig 8B, lanes 3 and 4), distinct from the impaired activation of PKR observed in VVΔE3L-infected *Mda5* ^*-/-*^ cells (lanes 1 and 2). Taken together, these results indicate that not only is PKR required for the MDA5-dependent activation of IRF3 and induction of IFNβ expression, but activation of its catalytic function is dependent on MDA5 during VVΔE3L, but not VSV, infection.

## Discussion

PKR is a key component of the antiviral response to a wide variety of viruses [69, 70]. This antiviral effect has been largely attributed to its well-characterized ability to phosphorylate and, thereby, inactivate eIF2α, leading to inhibition of translation of both viral and cellular RNA. As a consequence of eIF2α phosphorylation and the subsequent inhibition of translational initiation, PKR activation also leads to the formation of stress granules that appear to have antiviral function [71]. Early studies indicated that PKR may also play a direct role in IFN induction in response to pIC, a dsRNA mimetic [36, 49]; however, a number of more recent genetic and biochemical studies have suggested that PKR can be superfluous for IFN induction, at least in response to some viruses, throwing into question whether PKR acts as a sensor or an effector in the antiviral response. For instance, IFN induction by pIC or Newcastle disease virus (NDV) infection was intact in PKR-null mice *in vivo*, and an apparent *in vitro* deficiency could be reversed by IFN priming, suggesting that PKR-independent pathways mediated IFN induction [72]. Discovery of cytoplasmic dsRNA sensors, RIG-I, MDA5, and LGP2 and their associated signaling pathways, has provided a framework for understanding the primary pathways for innate immune responses to RNA viruses, without a documented role for PKR [73]. Nonetheless, experimental data have suggested that PKR may be a component of IFN production in response to some viruses, including West Nile virus [37], Theiler's murine encephalitis virus (TMEV), EMCV [38], measles virus [74] and Semliki Forest virus (SFV) [39]. Here, we provide evidence that PKR is involved in MDA5-mediated responses to virus infection and is necessary for IFN induction. Mechanistically, we found that MDA5 and PKR proteins associate in a complex, and that MDA5 augments virus-induced activation of PKR. These results suggest that that PKR participates as part of the viral sensor machinery in concert with MDA5 to recognize foreign RNA and induce IFN.

We reexamined the role of PKR in IFN induction by looking specifically at responses to VVΔE3L infection and other MDA5-mediated responses [42]. Consistent with earlier studies, the absence of PKR severely impaired IFN induction following pIC transfection [36, 49]. Additionally, the absence of PKR resulted in a significant inhibition of VVΔE3L-induced IFN production, a phenotype that strongly resembled that seen in MDA5-null cells [42]. This impaired IFN phenotype was not seen in response to FluΔNS1 or SeV, strong activators of RIG-I signaling [5]. In fact, IFNβ levels were increased following FluΔNS1 infection of PKR-null cells. This result likely reflects restoration of a protein synthesis-dependent positive feedback loop that would be impaired following PKR-mediated host translational shutoff [48, 75]. Interestingly, eIF2α S51A mutant cells, which also failed to arrest translation due to PKR activation, demonstrated a similar enhancement in FluΔNS1-mediated IFN production. However, increased IFN levels were also observed during FluΔNS1 infection in the presence of CHX, which also inhibits translation. However, it is likely that increased IFN following CHX treatment likely reflects stabilization of short-lived mRNA reported to occur under these conditions [76]. Furthermore, while loss of PKR abrogated MDA5-dependent IFN induction, mutation of eIF2α did not, suggesting that the requirement for PKR during IFN induction is not dependent on its ability to inhibit protein synthesis.

One of the antiviral functions of PKR is its role in formation of stress granules, which are triggered by translational stalling that occurs following eIF2α phosphorylation [77]. These structures, which have been associated with both proviral and antiviral phenotypes, form in the cytoplasm of cells following infection with a variety of different viruses, including VVΔE3L [78]. Formation of these granules is dependent on PKR and involves cytoplasmic helicases, including RIG-I, MDA5, and DHX36 [56, 58, 79, 80]. However, it is unlikely that the stress granule-inducing function of PKR explains its cooperation with MDA5 during activation of IRF3 and IFNβ induction. Consistent with previous reports, we observed that stress granule formation was dependent on the phosphorylation of eIF2α, probably due to the subsequent translational arrest. In our studies, stress granules formed following VVΔE3L infection in WT cells, but not in eIF2α-S51A cells, although IFN was induced in both genetic backgrounds. These data show that phosphorylation of this PKR substrate, translation inhibition, and subsequent stress granule formation were not prerequisites for IFNβ induction (Fig 3).

Although PKR has been implicated in IFN induction, the mechanism behind its involvement is not fully understood. Recently, it was reported that PKR enhances IFNβ production in response to a mutant form of measles virus [36, 74]. The requirement for PKR was linked to its role in the activation of NFκB [74] and was ascribed to translational shutoff [59], since the expression of mutant eIF2α S51A also resulted in inhibition of IFN induced by the mutant virus. However, results from experiments with other viruses demonstrated that PKR influences IFN production in response to EMCV and SFV independently of eIF2α [38], consistent with our data. Our results indicate that, like EMCV and SFV, PKR-dependent IFN induction in response to VVΔE3L is eIF2α phosphorylation-independent. These distinct roles observed for PKR likely reflect the differential requirements for its enzymatic activity that depend on the viral stimulus. Although MDA5 may play a moderate role in response to measles virus infection, RIG-I, rather than MDA5, is the predominant sensor during infection for this virus [81]. The role for PKR documented here appears to be exclusive for MDA5-activating viruses, and may be circumvented by redundant signaling following activation of RIG-I.

To address potential mechanisms of PKR action, we noted that the presence of PKR influences the activated state of IRF3 in response to VVΔE3L and EMCV. Consistent with the hypothesis that PKR is required only for MDA5 signaling, the absence of PKR did not impact the ability of SeV, a strong activator of RIG-I, to activate IRF3 (Fig 4). Interestingly, the ability of PKR to influence IRF3 activation observed here is distinct from a recent study that reported a PKR-dependent decrease in EMCV-induced IFN protein expression that correlated with a change in polyadenylated (poly(A)), but not total IFN mRNA levels [38]. These investigators ascribed the decrease in IFN protein levels to enhanced shortening of IFN mRNA poly(A) tails in the absence of PKR, resulting in impaired translation. However, the defect in IRF3 translocation documented here suggests that, in this alternative pathway, PKR is acting upstream of IFN transcription. Furthermore, we observed a loss of IFNβ mRNA transcript levels in the absence of PKR, regardless of poly(A) length (S6 Fig). Thus, the role of PKR in IFN production may be complex and involve multiple points of influence. Support for this notion comes from documented responses to SFV infection. Both IFNβ mRNA and protein levels are impaired in response to SFV infection in the absence of PKR. However, the impairment is more pronounced for IFNβ protein levels [39], suggesting that PKR exerts both a transcriptional and post-transcriptional influence on IFN production.

Consistent with our studies, Zhang and Samuel demonstrated that PKR, MAVS, and MDA5 are important for VVΔE3L-induced IRF3 activation [82], and reported a direct interaction between PKR and MAVS [83]. To address how PKR may influence the MDA5-MAVS-IRF3 pathway, we found that PKR physically interacted in a complex with MDA5 (Fig 5A, 5B and 5C). This association appears to occur independently of VVΔE3L infection, as both ectopic and endogenous MDA5 co-purified with PKR under mock as well as infected conditions. We also demonstrate in Fig 5B that the association between MDA5 and PKR is not bridged by RNA, since pre-treatment of extracts with nuclease had no effect on the interaction. It is, therefore, possible that under basal settings, PKR and MDA5 exist as a complex that must be further activated in response to virus infection. Whether the presence of viral RNA is detected by MDA5 or PKR, this activation event presumably requires PKR’s catalytic function, since PKR autophosphorylation was diminished in the absence of MDA5. Interestingly, an interaction between PKR and RIG-I was also detected, but only in response to VVΔE3L infection (Fig 5A). This result is consistent with a recently reported interaction between PKR and RIG-I following FluΔNS1 infection [79], and highlights a distinct mechanism of action between MDA5 and RIG-I in regards to their association with PKR. Taken together, it is tempting to speculate that interactions of PKR with RLH proteins may depend on activation of the cytoplasmic sensors. For instance, engagement of MDA5 with VVΔE3L during infection could lead to PKR-MDA5 interaction, while activation of RIG-I during FluΔNS1 infection could lead instead to PKR-RIG-I interaction. However, at least in our experiments, we saw no requirement for PKR activation during RIG-I signaling, suggesting that its involvement downstream of RIG-I may be redundant with other signals.

We further investigated PKR function by ectopically activating it as a GyrB.PKR fusion protein. Coumermycin-mediated activation of PKR was sufficient for IFNβ induction, accompanied by the activation and phosphorylation of IRF3. Highlighting the requirement for its catalytic function, this effect was no longer observed when an enzymatically inactive, GyrB.PKR (K296H) mutant was expressed. Moreover, direct activation of PKR with this approach displayed MAVS-, but not MDA5-dependency, suggesting that the requirement for PKR during VVΔE3L infection lies downstream of MDA5, but upstream of MAVS. However, the activation of endogenous PKR during VVΔE3L infection remained strictly dependent on MDA5. Stimulation of PKR catalytic activity was impaired in MDA5-deficient cells, suggesting that MDA5 augments the ability of PKR to be activated by dsRNA. Structural studies of PKR have demonstrated that it is activated through conformational changes and dimerization mediated by interactions between its amino-terminal dsRNA binding domains and pathogen-derived dsRNA species [84, 85]. Sufficient length requirements are necessary in order for these dsRNA species to induce PKR dimerization and activation [86]. However, recent studies have indicated that a variety of RNA structures can be recognized by PKR, allowing it to be activated by a diverse set of pathogenic signals [87, 88]. It is possible that MDA5, which also recognizes pathogen-derived dsRNA [89], augments the loading and/or activation of PKR by dsRNA, possibly by acting to remove RNA-binding proteins that would otherwise interfere with PKR activation [90] or facilitating the formation of RNA structures with sufficient stimulatory characteristics [88]. In this regard, it is interesting that it is the helicase domain of MDA5 that can augment PKR activation by stripping RNA-binding proteins from IFN-stimulatory RNA, becoming phosphorylated by PKR in the process [90]. This may also be the same domain that cooperates with PKR during VVΔE3L infection, since the isolated MDA5 CARD domains were able to act in a PKR-independent fashion (Fig 5F).

In conclusion, our data indicate that PKR is required for robust MDA5-dependent responses, and that MDA5 plays a role in PKR activation during viral infection. It remains to be determined how MDA5 facilitates PKR catalytic activity and how activated PKR augments the MAVS-dependent stimulation of IRF3 and IFNβ transcription. In particular, the direct PKR substrate responsible for signaling has not been identified. MDA5 is one likely candidate, since it has been shown to be a PKR substrate [90]. Similarly, PKR itself may be a relevant substrate, potentially stabilizing its interaction with MDA5 and/or MAVS. Indeed, PKR becomes multiply autophosphorylated following activation, although the function of the majority of these phosphorylation sites remains to be defined [91]. An attractive model, consistent with the studies reported here, would posit that MDA5, through its RNA helicase activity, facilitates transfer of viral dsRNA to PKR, enhancing its overall catalytic activity in infected cells. Similarly, PKR activity, either through phosphorylation of MDA5 or additional factors, contributes to MDA5 filament formation, a process that is structurally distinct from activated RIG-I and required for downstream signaling [90]. Altogether, the studies reported here describe a critical catalytic function for PKR that responds to viral infection in an MDA5-dependent manner to govern the IFN response. Further studies will be needed to more fully understand how cellular signaling pathways are wired to tailor the innate response to different classes of viral pathogens.

## Materials and Methods

### Cell lines

A549, HEK293T, HT1080-GyrB-PKR, HT1080-GyrB-PKR-K296H, and MEF cells were maintained in Dulbecco's modified Eagle's Medium (DMEM; Cellgro) complemented with 10% calf serum. A549 and HEK293T cells stably expressing short-hairpin RNA against either PKR (shPKR) or a nonspecific sequence (shNS) were maintained in the above media containing 5 ug/mL and 3 ug/mL of puromycin, respectively. MDA5 and RIG-I deficient and control MEFs were kindly provided by Shizuo Akira (Osaka University). IRF3 deficient MEFs were kindly provided from Tadasugu Taniguchi (University of Tokyo). HT1080-GyrB-PKR and HT1080-GyrB-PKR-K296H cells were a generous gift from Antonis Koromilas (McGill University), and eIF2α-S51A cells were a gift from Randall Kaufman (University of Michigan). MEFs have been isolated from two distinct strains of PKR-null mice. Cells used to conduct the majority of studies reported here were kindly provided by Bryan Williams (Monash University), and express a truncated form of the protein lacking the RNA binding domain [92]. Similar results showing the requirement for PKR for IFN production in response to VVΔE3L were also obtained with PKR-null MEFs kindly provided by John Bell, which express a truncated a form of the protein lacking the catalytic domain [93].

A549 and HEK293T cells stably expressing short-hairpin RNA were generated by MLP-shPKR and MLP-shNS retroviruses derived by co-transfection with EcoPac [94] and VSV-G (vesticular stomatitis virus glycoprotein) into HEK293T cells using the calcium phosphate precipitation method. Supernatant was harvested 36–48 h post transfection, clarified, and used for three sequential infection cycles of either A549 or HEK293T cells followed by selection with 5 ug/ml or 3ug/mL of puromycin, respectively.

pLKO.1-puro pseudotyped lentiviruses expressing hairpins against MDA5 and MAVS were packaged in HEK293T cells. Viral supernatants were used to transduce HT1080-GyrB-PKR cells followed by selection for hairpin expression with puromycin.

pLVX-mCherry VSV-G pseudotyped lentiviruses expressing RIG-I-CARD or MDA5 were packaged by co-transfection of HEK293T cells with psPAX2 and pMD2.G, gifts from Didier Trono. Viral supernatant was harvested at 48 and 72 h post transfection and concentrated by centrifugation at 25,000 rpm for 90 minutes. A549 shPKR and A549 shNS cells were subjected to three rounds of infection with concentrated virus and the resulting cells were assayed for IFNβ production by real time PCR and mCherry fluorescence by microscopy after 48 h.

### Plasmids

The PKR retroviral short hairpin RNA (shRNA) vector contained a previously described hairpin sequence [95] within a mir-30 backbone (5'-TGCTGTTGACAGTGAGCGAgcagggagtagtacttaaataATAGTGAAGCCACAGATGTATtatttaagtactactccctgcCTGCCTACTGCCTCGGA-3'), which was cloned into the MSCV-based retroviral vector MLP. pCAGGs constructs expressing full-length RIG-I and MDA5, kind gifts from Christopher Basler (Icahn School of Medicine at Mount Sinai), were cloned from their respective vectors into the lentiviral vector pLVX-mCherry. Constitutively active RIG-I (pEF-BOS-RIG-I-MIII) was the kind gift of Curt Horvath (Northwestern University) [65].

pLKO.1-puro constructs expressing shRNA against MDA5 (CCGGCCAACAAAGAAGCAGTGTATACTCGAGTATACACTGCTTCTTTGTTGGTTTTTG) and MAVS (CCGGCAAGTTGCCAACTAGCTCAAACTCGAGTTTGAGCTAGTTGGCAACTTGTTTTTTG;) were provided by the NYU School of Medicine shRNA Core Facility [96]. The Flag-tagged STAT2 construct, pLpC-Flag-Stat2 was previously described [64]. An expression construct for MDA5-CARD was generated by cloning the first 267 amino acids of human MDA5 into pLpC

### Viruses and infections

Vaccinia Virus ΔE3L (VVΔE3L) derived from the Western Reserve strain was a kind gift from Bertram Jacobs (Arizona State University). Sendai Virus (SeV), Cantell strain was purchased from Charles River. The VSV strain (VSV-GFP M51R) used in these studies was a gift from Benjamin tenOever (Icahn School of Medicine at Mount Sinai), and contains a mutation in the matrix viral protein (M51R) that renders the virus incapable of inhibiting IFN production [97]. This mutant was used as a positive control for a RIG-I ligand, and as a robust stimulator of type I IFN production. Influenza A ΔNS1 (Flu ΔNS1) was a gift from Adolfo Garcia-Sastre (Icahn School of Medicine at Mount Sinai), and encephalomyocarditis virus (EMCV) was a gift from Jan Vilcek (NYU School of Medicine).

Unless otherwise indicated, cells were infected at a multiplicity of infection (MOI) of approximately 3, with the exception of FluΔNS1, which was used at an MOI of 1. SeV was used at 200 HA units/ml. For all infections, virus was diluted in serum-free DMEM and incubated with target cells at 37°C for 1 h, followed by replacement with standard growth medium. Unless otherwise indicated, virus infected cells were assayed at 8 h post infection.

### Treatments and transfections

PKR inhibitor (PKR-I; Calbiochem) was added to cells at the indicated concentrations and incubated for 1 h. Following incubation, the inhibitor was removed and cells were infected as indicated. Cycloheximide (CHX) was added to cells at 50 ug/ml for the final 4 h of viral infection. Cells were treated with either 100 ng/ml or 200 ng/mL of coumermycin (Sigma) for 8 or 24 h, or with 5 ng/ml IFNγ (Amgen Biologicals) for 8 h.

For polyriboinosinic:polyribocytidylic acid (pIC) transfections, cells were transfected with 2 ug pIC (Invivogen) using Lipofectamine 2000 (Invitrogen) and incubated for 4 h at 37°C before RNA extraction.

### Luciferase assay

HEK293T cells were seeded onto 12-well dishes and transfected using the calcium phosphate method with 1 ug of firefly luciferase plasmid fused to human IFNβ promoter, 1.4 ug of plasmids expressing β-galactosidase or renilla luciferase, and 4 ug of plasmids expressing RIG-I-CARD (pLVX-RIG-I-CARD or pCDNA3- RIG-I-CARD), MDA5-CARD (pLpC- MDA5-CARD), RIG-I-MIII (pEF-Bos-Flag-RIG-I-MIII), or MDA5 (pLVX-MDA5). Cells were harvested 48 h post transfection and assayed for firefly luciferase and β-galactosidase or renilla luciferase activity. Firefly luciferase levels were normalized to β-galactosidase or renilla luciferase activity and experiments were performed in triplicate.

### Quantitative real time PCR (qRT-PCR)

RNA isolated with TriZol (Invitrogen) was used to generate cDNA with Moloney murine leukemia virus (M-MLV) reverse transcriptase (Invitrogen), according to the manufacturer's instructions. While oligo dT was primarily used to prime cDNA transcripts for qRT-PCR, random hexamers were used for S6 Fig to demonstrate the loss of IFNβ in infected *Pkr* ^*-/-*^ MEFs was not due to poly(A) trimming. Transcripts were then quantified by qRT-PCR with SYBR green (Molecular Probes) using the following primers:

human GAPDH: F 5' TGGAAGGACTCATGACCACA 3' and R 5' TTCAGCTCAGGGATGACCTT 3'; human IFNβ: F 5' GTCTCCTCCAAATTGCTCTC and R 5' ACAGGAGCTTCTGACACTGA 3'; human GBP1: F 5' TTCTTCCAGATGACCAGCAG 3' and R 5' GCTAGGGTGGTTGTCCTTGA 3'; mouse GAPDH: F 5' TGAGGACCAGGTTGTCTCCT 3' and R 5' CCCTGTTGCTGTAGCCGTAT 3'; mouse IFNβ: F 5' CCCTATGGAGATGACGGA 3' and R 5' CTGTCTGCTGGTGGAGTTC 3'; human TNFα: F 5’ CCTGACATCTGGAATCTGGAGACC 3’ and R 5’ CTGGAAACATCTGGAGAGAGGAAGG 3’; human IL1β: F 5’ GGGCCTCAAGGAAAAGAATC 3’ and R 5’ TTCTGCTTGAGAGGTGCTGA 3’; EMCV 3D: F 5’ GACGCTTGAAGACGTTGTCTTCTTA 3’ and R 5’ CCCTACCTCACGGAATGGGGCAAAG 3’; VV HA: 5’ CATCATCTGGAATTGTCACTACTAAA 3’ and R 5’ ACGGCCGACAATATAATTAATGC 3’; SeV NP: 5’ TGCCTGGAAGATGAGTTAG 3’ and 5’ GCCTGTTGGTTTGTGGTAAG 3’. Relative expression was determined by comparison to a standard curve generated from serial dilutions of cDNA containing abundant target sequences and normalized to the expression of GAPDH. Data were represented as the IFNβ/GAPDH ratio and the mean ± standard deviation between triplicate samples of a representative experiment were shown. Significance was determined by an unpaired t-test. Data shown are representative of at least three independent experiments.

### Immunoprecipitation and western blot analysis

Whole cell extracts were lysed in 1% NP-40 buffer (50 mM Tris, pH 7.5, 150 mM NaCl, 30 mM NaF, 5 mM EDTA, 10% glycerol, 1% NP-40, 1 mM PMSF, 1 mM Sodium Orthovanadate, protease inhibitor) for 20 min on ice before centrifugation at 4°C for 10 min. Protein concentrations were quantified by Bradford assay. For immunoprecipitations (IP) in Fig 5A, HEK293T cells were transfected with FLAG-tagged RIG-I, MDA5, E3L or STAT2 using the calcium phosphate precipitation method (Fisher Scientific). Thirty-six hours post-transfection, cells were infected with VVΔE3L at an MOI of 10 for 8 h, followed by lysis in 1% NP-40 buffer. IPs were performed with 800ug of total protein and incubated with anti-FLAG M2 affinity gel (Sigma-Aldrich) overnight at 4°C. For Fig 5B, lysates from transfected HEK293Ts were treated with 100 units/mL of micrococcal nuclease (Affymetrix USB) for 30 min at 37°C prior to incubation with incubation with beads. Beads were washed four times with RIPA buffer (50 mM Tris pH 7.8, 150 mM NaCl, 0.1% SDS, 0.5% sodium deoxycholate, 1% Triton-X-100) supplemented with protease inhibitor cocktail (Sigma-Aldrich), 1 mM PMSF, 100 mM sodium fluoride, 2 mM sodium pyrophosphate and 2 mM sodium orthovanadate), resuspended in Laemmli buffer, and boiled at 95°C for 5 min. Additionally, 5% of total protein lysate was included as input control. For IPs in Fig 5C, A549 cells were first treated with 150U/ml of human IFNα 2a for 14 hours to induce detectable levels of MDA5 and lysed in 1% NP-40 buffer. Protein A agarose beads (Thermo Scientific) were blocked with 5% BSA+ PBS prior to incubation with 2 mg of lysates overnight at 4°C. 5% input and IP reactions were resolved by 8% SDS-PAGE. For isolation of cytoplasmic and nuclear fractions, cells were initially lysed in RSB (10 mM Tris pH.7.4, 10 mM NaCl, 3 mM MgCl_2_, 0.5 mM DTT, 1 mM sodium orthovanadate, 1 mM PMSF, and protease inhibitor cocktail (Biotool) for 20 min on ice with occasional vortexing. Following incubation, cells were centrifuged at 7,500 rpm for 5 min at 4°C. Supernatant was retained as the cytoplasmic fraction. The remaining nuclear pellet was washed with 1xPBS and lysed in Extraction Buffer (20 mM HEPES pH. 7.9, 420 mM NaCl, 1.5 mM MgCl_2_, 0.2 mM EDTA, 25% glycerol, 0.5 mM DTT, 1 mM sodium orthovanadate, 1 mM PMSF, and protease inhibitor cocktail (Biotool) for 30 min on ice with occasional vortexing. Cells were centrifuged at 14,000 rpm for 20 min at at 4°C. Supernatant was retained as nuclear fraction.

Samples were resolved by 10% SDS-PAGE, transferred to PVDF membranes (Millipore) and blocked for 1 h in 5% non-fat milk + TBS (20 mM Tris-HCl pH 7.5, 150 mM NaCl). Membranes were probed with primary antibodies diluted in 5% BSA+TBS-0.1% Tween-20 buffer overnight at 4°C: anti-PKR (Santa Cruz; SC-707 for human, Cell Signaling; D7F7 for human, 1:1000, SC-1702 for murine, 1:1000), anti-Flag M2 (Sigma-Aldrich, 1:1000), anti-eIF2α-pSer51 (Cell Signaling, 1:1000), anti-eIF2α (Cell Signaling, 1:1000), anti-phosphoIRF3 (Cell Signaling, 1:1000), anti-IRF3 (Zymed, 1:2000), anti-Histone 3 (Abcam, 1:1000), anti-human IRF3 (Pharmingen, 1:1000), anti-p38α MAP kinase (Cell Signaling 5F11, 1:2000), anti-MDA5 (Cell Signaling, D74E4, 1:1000) and anti-Actin (Chemicon International, 1:1000). Antibodies against viral products VV-I3L and VSV-G were kind gifts from Ian Mohr and Benjamin tenOever, respectively. Membranes were probed with HRP-linked goat anti-mouse IgG (KPL; 1:5000) or goat anti-rabbit IgG (Thermo Scientific; 1:5000) and developed with enhanced chemiluminescence (ECL; Millipore).

### Immunofluorescence

WT eIF2α and mutant eIF2α S51A MEFs were seeded onto 8-well chamber slides (Millipore, EZ Slides) and infected with VVΔE3L at MOI = 1. At 8 hpi, cells were fixed with 4% Formaldehyde for 1 hr at room temperature (RT), followed by two washes with PBS, and permeabilization with cold methanol for 5 min. Cells were rinsed twice in PBS before incubation with Blocking buffer (5 mg/mL BSA and 0.04% Tween 20 in PBS) for 30 min at RT. Cells were overnight at 4°C incubated in primary anti-G3BP1 (BD Biosciences, #611126), prepared in Blocking buffer at 3 ug/mL. After three washes in PBS, cells were incubated in anti-mouse secondary (Life Sciences, AlexaFluor 588) at 1:200 in Blocking buffer for 1hr at RT. This was followed by three additional washes in PBS and incubation with DAPI at 1:1000 in PBS for 1min. Finally, cells were washed five times in PBS before mounting (Vectashield). G3BP1 and DAPI expression was visualized using the Nikon DS-Qi1 inverted fluorescent microscope.

### *In vitro* kinase assay

PKR catalytic activity was assayed as previously described [98]. In brief, infected cells were lysed in IP lysis buffer (50 mM Tri-HCl pH 7.6, 150 mM NaCl, 10% glycerol, 1% NP-40, 5 mM EDTA, 1 mM DTT, 100 mM NaF, 2 mM sodium pyrophosphate, 2 mM sodium orthovanadate, 1 mM PMSF). Equal amounts of protein were incubated with anti-PKR antibody (Santa Cruz) for 30 min on ice and then incubated with protein A agarose (Thermo Scientific) overnight at 4°C. The beads were washed twice in IP lysis buffer and twice in DBGA (10 mM Tris-HCl pH 7.6, 50 mM KCl, 2 mM MgOAc, 20% glycerol, 7 mM beta-mercaptoethanol). The beads were resuspended in 50 μl DBGB (DBGA containing 2.5 μM MnCl_2_) followed by the addition of 5 μl ATP mix [10 μCi [γ-^32^P]ATP (6000Ci/mmol), 0.5 μl 100 μM ATP, 3.5 μl DBGA]. The mixture was incubated at 30°C for 30 min, and the beads were resuspended in 1X Laemmli buffer and boiled. Samples were resolved by 10% SDS-PAGE and visualized by phosphorimager analysis.

## Supporting Information

S1 Fig Knockdown of PKR expression with short-hairpin RNA. (A) Western blot analysis on A549 cells stably expressing short-hairpin RNA (shRNA) against non-specific (NS) RNA and PKR. Lysates were probed for indicated protein expression. (B) Densitometry was performed on S1A Fig to quantify PKR expression levels. Related to Fig 2C.
(TIF)

S2 Fig Quantification of viral infection in *Pkr* ^*+/+*^ and *Pkr* ^*-/-*^ MEFs. Viral load 8 hpi was determined by qRT-PCR for viral RNA. (A) Quantification of vaccinia virus HA gene. (B) Quantification of EMCV 3D gene. (C) Quantification of SeV NP gene. Related to Fig 4.
(TIF)

S3 Fig Ectopic expression of RIG-I and MDA5. A549 cells expressing NS shRNA or PKR shRNA were infected with IRES-mCherry lentiviruses expressing empty vector, RIG-I-CARD, or MDA5, as indicated. Relative infection was determined by fluorescent microscopy for mCherry expression. Related to Fig 5D.
(TIF)

S4 Fig Knockdown of PKR expression with short-hairpin RNA. (A) Lysates from HEK293T cells stably expressing shRNA against either NS RNA or PKR were analyzed for the indicated protein expression. (B) Densitometry was performed on (A) for PKR expression. Related to Fig 5E and 5F.
(TIF)

S5 Fig Induction of TNFα and IL1β by conditionally active PKR. Real-time PCR analysis of TNFα (A) and IL1β (B) expression in HT1080.GyrB.PKR cells following treatment with 200ng/mL coumermycin.
(TIF)

S6 Fig IFNβ induction during VVΔE3L infection is abrogated in *Pkr* ^*-/-*^ MEFs. Real time-PCR analysis of IFNβ expression following random priming of RNA from *Pkr* ^*+/+*^ and *Pkr* ^*-/-*^ MEFs infected with VVΔE3L showed that absence of IFN expression was not explained by loss of poly(A) tails.
(TIF)
