# Supplementary figures and images for: PKR Transduces MDA5-Dependent Signals for Type I IFN Induction
Source: PLoS Pathog. 2016 Mar 3;12(3):e1005489. doi: 10.1371/journal.ppat.1005489 (PMC4777437; doi:10.1371/journal.ppat.1005489)

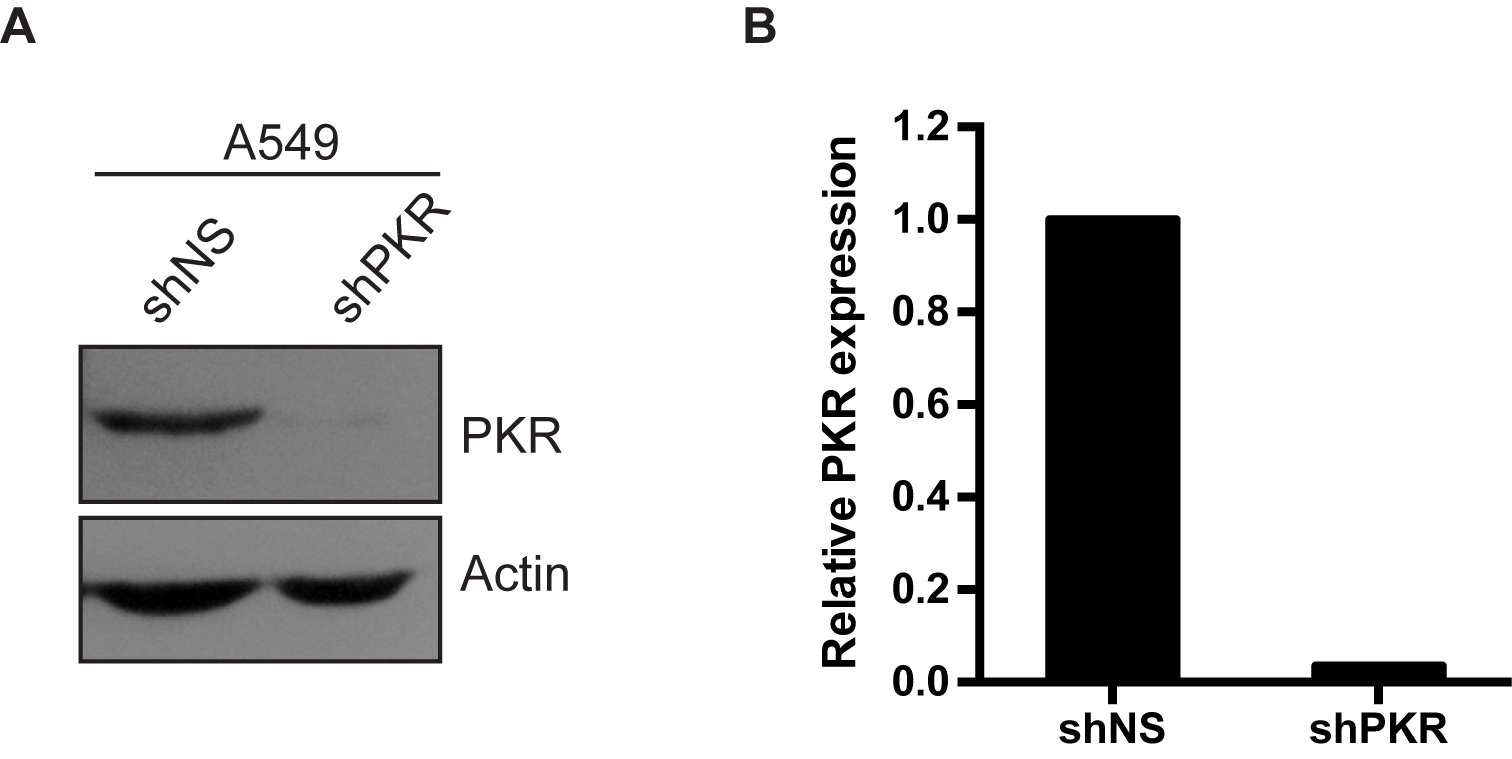

Supplement: S1 Fig — (A) Western blot analysis on A549 cells stably expressing short-hairpin RNA (shRNA) against non-specific (NS) RNA and PKR. Lysates were probed for indicated protein expression. (B) Densitometry was performed on S1A Fig to quantify PKR expression levels. Related to Fig 2C. (TIF) [file ppat.1005489.s001.tif]

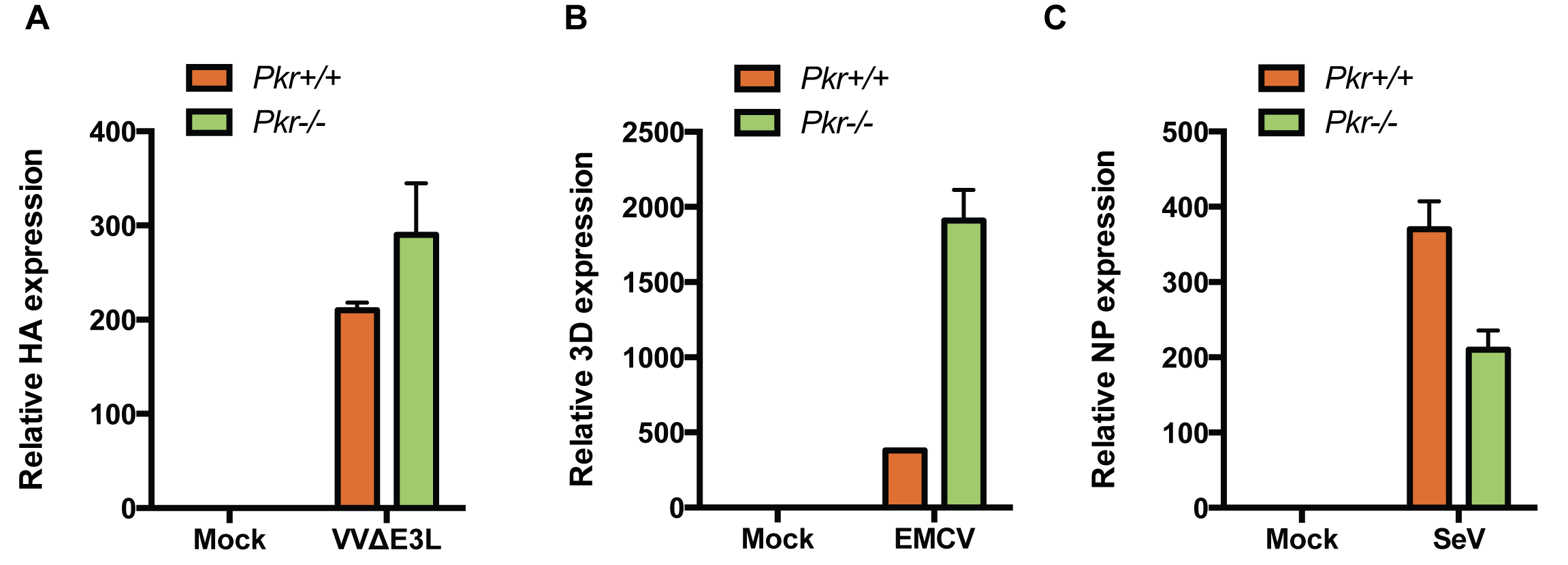

Supplement: S2 Fig — Viral load 8 hpi was determined by qRT-PCR for viral RNA. (A) Quantification of vaccinia virus HA gene. (B) Quantification of EMCV 3D gene. (C) Quantification of SeV NP gene. Related to Fig 4. (TIF) [file ppat.1005489.s002.tif]

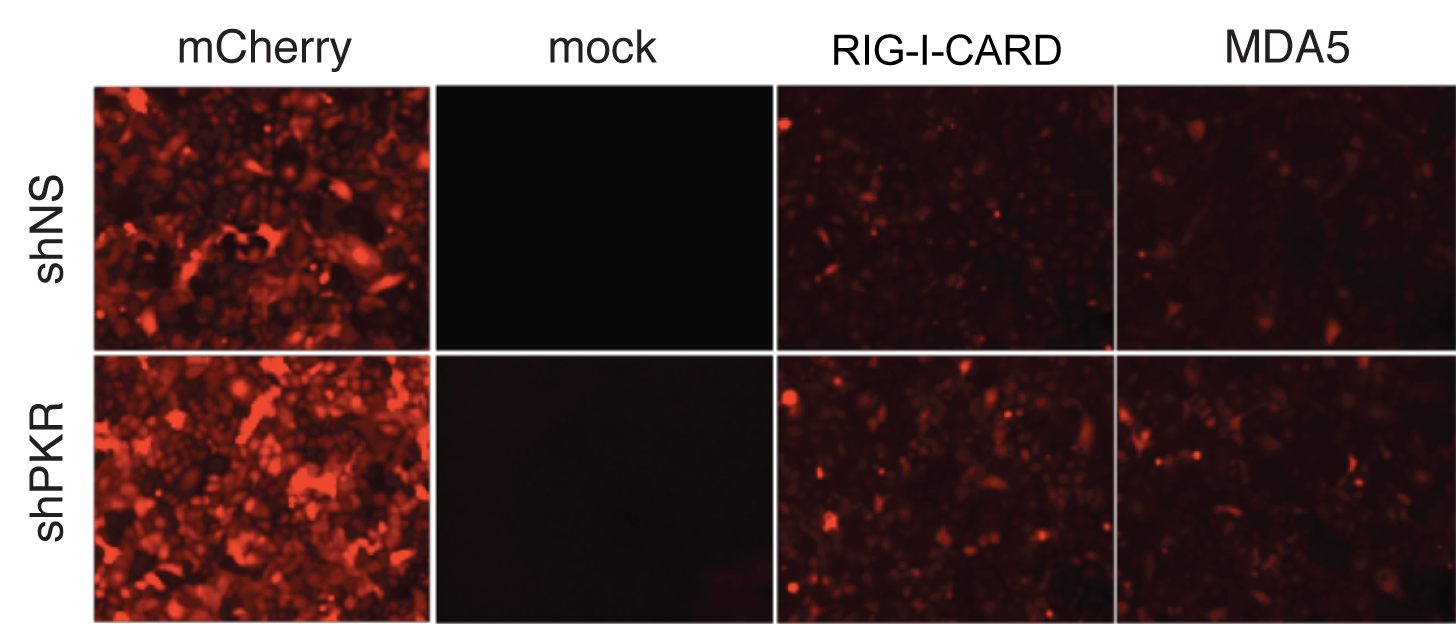

Supplement: S3 Fig — A549 cells expressing NS shRNA or PKR shRNA were infected with IRES-mCherry lentiviruses expressing empty vector, RIG-I-CARD, or MDA5, as indicated. Relative infection was determined by fluorescent microscopy for mCherry expression. Related to Fig 5D. (TIF) [file ppat.1005489.s003.tif]

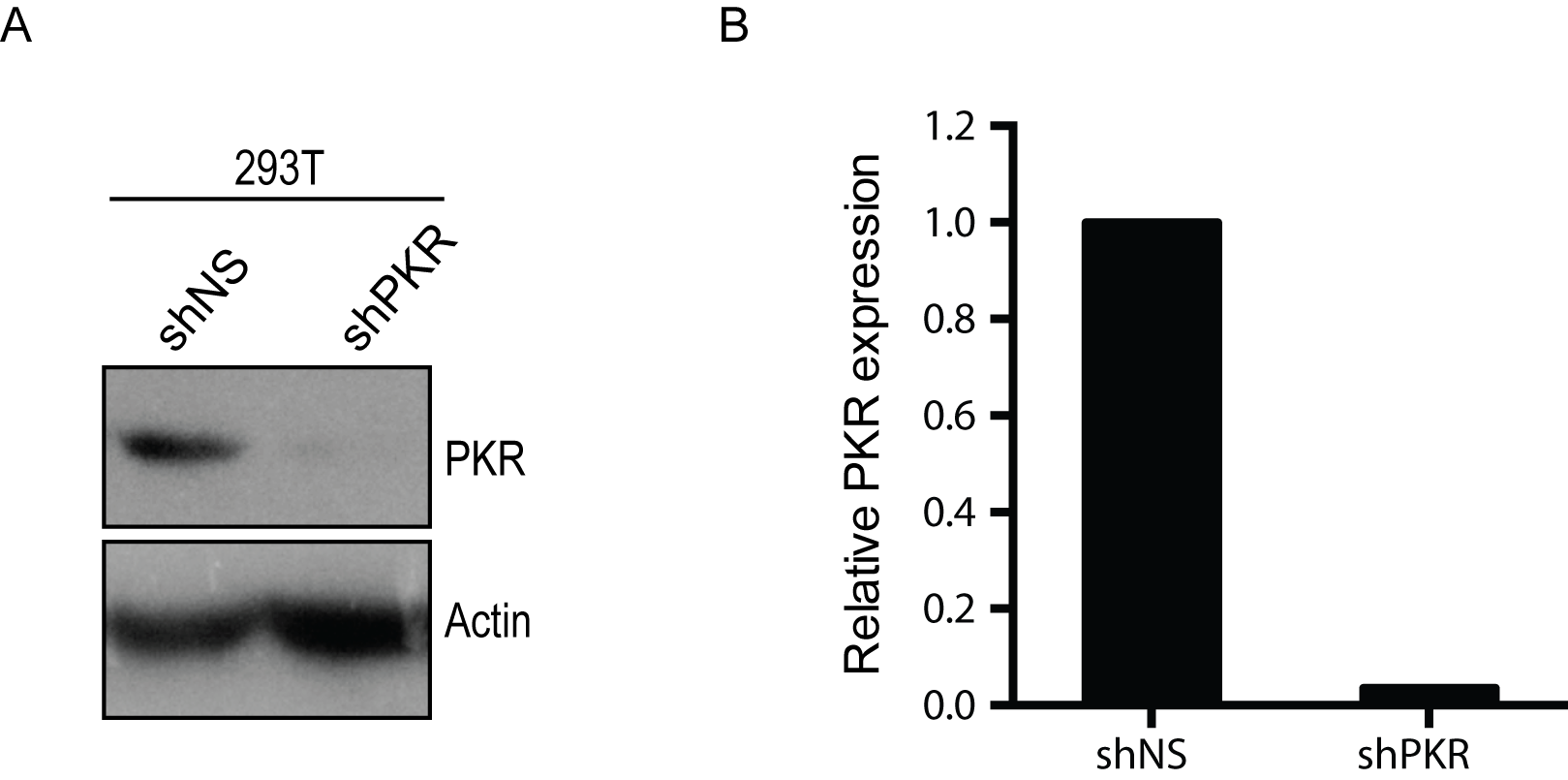

Supplement: S4 Fig — (A) Lysates from HEK293T cells stably expressing shRNA against either NS RNA or PKR were analyzed for the indicated protein expression. (B) Densitometry was performed on (A) for PKR expression. Related to Fig 5E and 5F. (TIF) [file ppat.1005489.s004.tif]

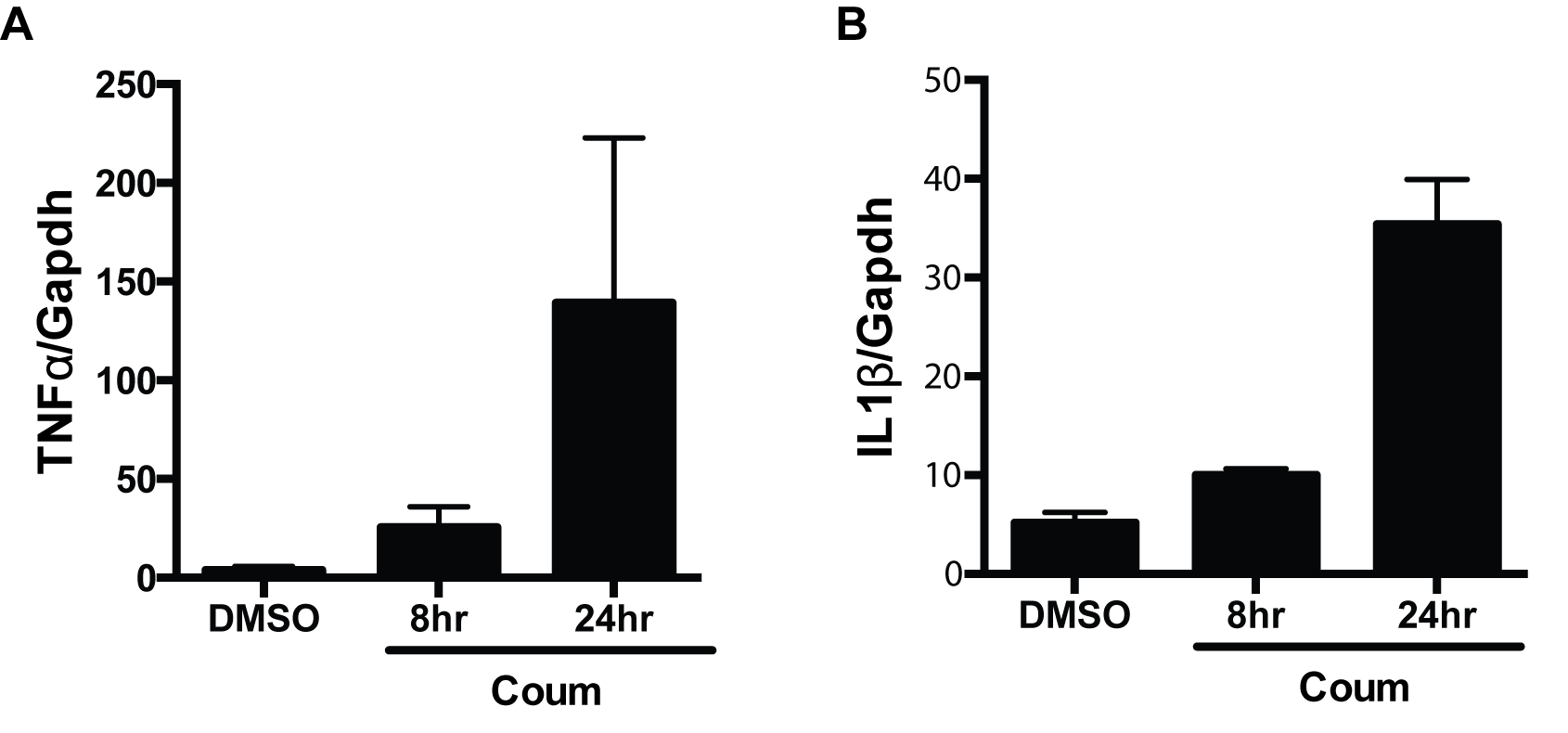

Supplement: S5 Fig — Real-time PCR analysis of TNFα (A) and IL1β (B) expression in HT1080.GyrB.PKR cells following treatment with 200ng/mL coumermycin. (TIF) [file ppat.1005489.s005.tif]

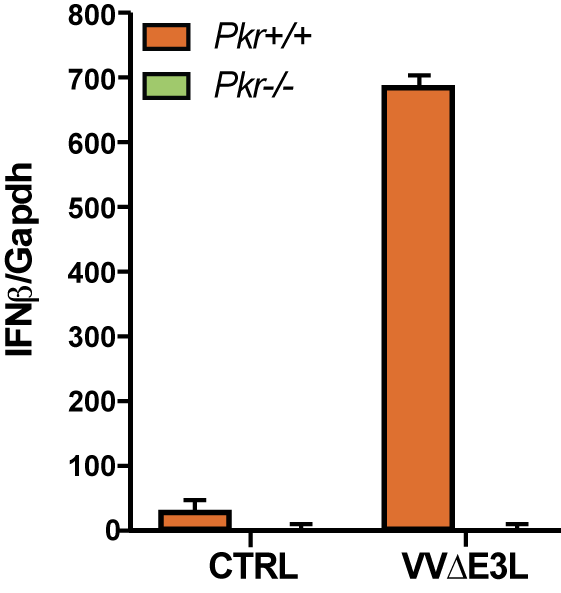

Supplement: S6 Fig — Real time-PCR analysis of IFNβ expression following random priming of RNA from Pkr +/+ and Pkr -/- MEFs infected with VVΔE3L showed that absence of IFN expression was not explained by loss of poly(A) tails. (TIF) [file ppat.1005489.s006.tif]
